# Effect of Cytosolic pH on Inward Currents Reveals Structural Characteristics of the Proton Transport Cycle in the Influenza A Protein M2 in Cell-Free Membrane Patches of *Xenopus* oocytes

**DOI:** 10.1371/journal.pone.0107406

**Published:** 2014-09-11

**Authors:** Mattia L. DiFrancesco, Ulf-Peter Hansen, Gerhard Thiel, Anna Moroni, Indra Schroeder

**Affiliations:** 1 Department of Biosciences and CNR-IBF, University of Milan, Milan, Italy; 2 Plant Membrane Biophysics, Technical University of Darmstadt, Darmstadt, Germany; 3 Department of Structural Biology, University of Kiel, Kiel, Germany; Zhejiang University, China

## Abstract

Transport activity through the mutant D44A of the M2 proton channel from influenza virus A was measured in excised inside-out macro-patches of *Xenopus laevis* oocytes at cytosolic pH values of 5.5, 7.5 and 8.2. The current-voltage relationships reveal some peculiarities: 1. “Transinhibition”, i.e., instead of an increase of unidirectional outward current with increasing cytosolic H^+^ concentration, a decrease of unidirectional inward current was found. 2. Strong inward rectification. 3. Exponential rise of current with negative potentials. In order to interpret these findings in molecular terms, different kinetic models have been tested. The transinhibition basically results from a strong binding of H^+^ to a site in the pore, presumably His37. This assumption alone already provides inward rectification and exponential rise of the IV curves. However, it results in poor global fits of the IV curves, i.e., good fits were only obtained for cytosolic pH of 8.2, but not for 7.5. Assuming an additional transport step as e.g. caused by a constriction zone at Val27 resulted in a negligible improvement. In contrast, good global fits for cytosolic pH of 7.5 and 8.2 were immediately obtained with a cyclic model. A “recycling step” implies that the protein undergoes conformational changes (assigned to Trp41 and Val27) during transport which have to be reset before the next proton can be transported. The global fit failed at the low currents at pH_cyt_ = 5.5, as expected from the interference of putative transport of other ions besides H^+^. Alternatively, a regulatory effect of acidic cytosolic pH may be assumed which strongly modifies the rate constants of the transport cycle.

## Introduction

M2 is a homo-oligomeric protein from the membrane of the influenza virus A with ion transport activity. It is well established that the transport of H^+^ by the M2 protein is a critical step in the infective cycle of the virus [1], [2]. After endocytosis by the host cell, the virus particle is transported into the endosome. In this acidic environment, the viral membrane fuses with the membrane of the endosome, and an acid-stimulated opening of the M2 channel catalyzes influx of H^+^ into the virus particle [3], [4].

Influx of protons causes uncoating of the viral RNP (ribonucleoprotein) and release into the host cell [5], [6]. From a pharmacological point of view, the interest in the M2 protein arises from the fact that a block of this channel can inhibit viral replication in host cells. Amino-adamantyls [7] or related derivatives [8] are inhibitors of the M2 channel, and they were already successfully used to terminate RNA replication of influenza A [9], [10].

**Figure 1:**
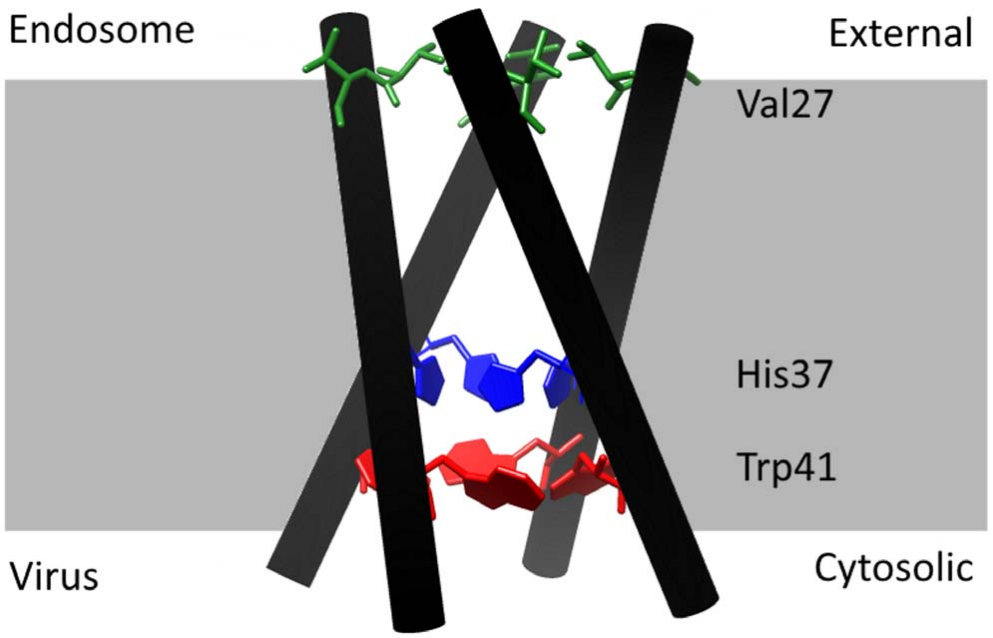
Structure of the M2 protein and its orientation during infection. Coordinates were taken from [15], pbd access code 3lbw. The image was created with the UCSF Chimera package [67], which is developed by the Resource for Biocomputing, Visualization, and Informatics at the University of California, San Francisco (supported by NIGMS P41-GM103311). In the present experimental setting, in which the M2 protein is expressed in *Xenopus* oocytes, the endosomal side corresponds to the external medium and the side facing to the interior of the virus particle corresponds to the “cytosolic” side. The critical residues Val27 (green), His37 (blue) and Trp41 (red) are shown explicitly.

The mechanism of conduction has been studied for a long time. Early models suggested a continuous water wire with His37 acting as a gate [11] or alternatively a shuttle mechanism which originated from the role of protonation and deprotonation of His37 [12]. In the meantime, overwhelming evidence has been compiled for the shuttle mechanism as reviewed by Hong and DeGrado [13]. The M2 protein is a tetramer with the N-terminus at the endosomal (external) side and the C-terminus at the virial (internal) side. The four helices form a pore for proton conduction with five layers of side chains as revealed by different structural studies (e.g. [14]–[16]): 1. Val27-gate (or valve) at the external N-terminal end of the pore, 2. the cavity lined by Ala30, Ser31 and Gly34, 3. the His37-box, 4. the Trp41-basket (or-gate), and 5. the Asp44/Arg45-box at the internal, C-terminal end of the pore. The layers important for the interpretation of our data are shown in Fig. 1.

Proton conduction between the layers is mediated by water wires and by intercalated water clusters [15]. In the cavity adjacent to the Val27-valve, crystal structure analysis reveals a region of diffuse density, suggesting dynamically or statistically disordered solvent. The rest of the pathway towards the internal side shows ordered clusters of water starting with the entry cluster on the N-terminal side of the His37-box. This cluster consists of 6 water molecules forming a dimer on top of 4 waters which are H-bonded to the Nδ of His37 and the backbone carbonyl residues of Gly34. In the His37-box, there is no direct H-bonding between the imidazoles of the four His37. Instead, they are connected by the highly structured network of water molecules. On the C-terminal side of the His37-box, the His37/Trp41 bridging cluster consisting of two waters provides the connection to the Trp41-basket. The waters are H-bonded to each Nε of His37. The Trp41-basket forms a gate for proton exchange between the viral interior and the His37-box. It is connected to Asp44 by 4 waters which form H-bonds between the indole NH of Trp41 and a carboxylate oxygen of Asp44. Finally, at the exit to the viral interior, there is a poorly ordered fifth water on the C-terminal side of the four waters. This structure is the basis for the model described below.

The His37-box has been called the “heart of the conduction mechanism” (e.g. [15], [17], [18]) as the control of proton transport through M2 depends on the imidazole moieties of His37. They have a double function as accepting and storing protons from the water molecules in the pore and regulating the open state of the Trp41/Asp44-box. Many studies based on different techniques [17], [19]–[22] have shown that the first two protons are bound to the Hi37-box with a high pKa of 8.2. The third one has a pKa of about 6 and the fourth of about 4 to 5.

Up to two protons, the Trp41-basket is closed, thus preventing the exchange of protons between His37 and the viral interior. The related backbone conformation has been revealed by crystal structure analysis [14], [15], ssNMR [23], solution NMR [24] and MD simulations [16]. At higher protonation states (+3 and +4), the Trp41-basket opens as has been shown by crystal structure analysis [14], [15]. This is in agreement with studies based on EPR spectroscopy [25], ssNMR [23], NMR [19], [26] and MD simulations [16], [19].

The closed state of the Trp41-basket strongly depends on the interaction with Asp44 [19] as also known from mutational studies [27]. The closed state is stabilized by direct or water-mediated H-bonds between Asp44 and Trp41 [15], [19], [24]. Drugs like rimantadine further stabilize this closed state by H-bonds between Asp44 and the drug, thus preventing opening of the proton pathway during infection [26], [27]. Mutations of Asp44 destabilize the closed state, leading to higher open probability and higher conductance [19]. The interaction between His37, Trp41 and Asp44 is responsible for the asymmetry of the flux/pH relationship as found in the high conductivity at acidic external pH, but not at acidic internal pH. The involvement of Trp41 was indicated by the finding that mutations of Trp41 (W41F) destroy the asymmetry by allowing access of protons to His37 from the inside [28].

Details of the protonation-induced destabilization of the C-terminal end of the pore are as follows: The helical kink near Gly34 related to the closed state is straightened upon increasing His37 protonation thus causing C-terminal dilation. The degree of dilation is not clear because of different findings in different structures [14]–[16], [29]. Nevertheless, the dilation is sufficient for the following effects: 1. Increase in the degree of hydration in the pore, 2. Decrease of electrostatic repulsion between multiple charged His37, 3. Opening of the Trp41-gate [19].

Acharya et al. [15] compared the Trp41-gate with the inner gate of K^+^ channels [30], [31]. However, there is one striking difference, which is important for the analysis here. NMR studies showed that the opening occurs in the micro- to millisecond range in M2 [24] and in K^+^ channels. However, because of the high transfer rates in cation channels, 10^4^ to 10^6^ ions/s can pass during one opening event of about 10 ms. In M2 with low transfer rates of 100 to 400 ions/s [32], it can be just a few ones, maybe, only one [15], [33]. Even more important than the time scale is the coupling between opening and permeant ion by His protonation in M2. This is not a mechanism which applies for the inner gate of K^+^ channels. Such a coupling between permeant ion and gating is supposed to occur in the selectivity filter of K^+^ channels like Kcv or BK leading to extremely fast gating in the microsecond or submicrosecond range [34]–[36]. Besides the strong selectivity, this, too, may be a basis to compare the His37-box of M2 with the selectivity filter of K^+^ channels.

Val27 at the external side acts as a second gate [26], [37], [38]. Crystal structure analysis [15] suggests that the bending of the transmembrane helices as controlled by the protonation state of the His37-box also extends to the Val27-gate in such a way that the gate closes when the Trp41-gate opens upon protonation. However, these results are not generally accepted as the large backbone motions were not found in MD simulations [38] or magic-angle spinning ssNMR experiments [22].

Kinetic models should explain the physiological behavior of channels on the basis of the structure. Modeling of measured proton transport has mainly been done with respect to the effect of external pH because this can be controlled easily in experiments using oocytes, mammalian cells or liposomes [4], [39]–[44]. In experiments dealing with internal pH, proton concentration was modified by loading via the pipette in whole-cell experiments on mammalian cells [4], [44] or by incubating *Xenopus* oocytes in acidic medium for a prolonged time [40]. Here, we present experiments on excised inside-out patches from oocytes, which enable a reliable control of internal pH. Even though the creation of stable macro-patches is cumbersome, we have obtained current-voltage curves (IV curves) at internal pH = 8.2, 7.5 and 5.5 which can be used to check whether the basic features of the above structural model are in line with the measured IV curves.

Some kinetic models applied so far to the proton conduction by M2 incorporated the existence of different protonation states of His37 [33], [45]. Whereas the previous models considered transitions between separate transport schemes for different protonation states, we try to account for the finding that the conformational changes are in the same time scale as the transitions of single protons [15], [24], [33]. Thus, we test whether the measured dependence of inward current on voltage and internal proton concentration is in agreement with a model where the protonation-induced opening of the Trp41-gate and the action of the Val27-gate are part of one kinetic cycle of transport.

## Materials and Methods

### Oocyte expression

D44A-A/M2-pGEM3 mutant cDNA derived from the influenza A/Udorn/72 strain was kindly provided by L. H. Pinto (Department of Neurobiology and Physiology, Northwestern University, Evanston, USA). cDNA was linearized with HindIII restriction enzyme, and cRNA was transcribed in vitro using a T7 RNA polymerase (Promega Corporation, Madison, USA). *Xenopus laevis* oocytes were purchased from EcoCyte Bioscience (Castrop-Rauxel, Germany) and RNA or RNAse-free water was injected (40 ng/oocyte), prepared according to standard methods [46]. Oocytes were incubated at 19°C in an ND96 solution containing (mM) (NaCl 96, KCl 2, CaCl_2_ 1.8, MgCl_2_ 1, HEPES 5, adjusted to pH 7.4 with NaOH), and moved to the same ND96 solution, adjusted to pH 8.2, after 24 h. Two-Electrode-Voltage-Clamp (TEVC) measurements were performed the day after injection, and macro-patch experiments were performed 2 to 7 days after injection.

### Electrophysiology

M2D44A expression level was monitored by recording oocyte currents with the TEVC technique (Geneclamp 500; Molecular Devices, Sunnyvale, USA). Electrodes were filled with 3 M KCl and had a resistance of 0.4 to 0.8 MΩ in 50 mM KCl. The oocytes were perfused at room temperature with a Barth’s solution containing (mM): NaCl 88, KCl 1, NaHCO_3_ 2.4, NaNO_3_ 0.3, CaCl_2_ 0.71, MgSO_4_ 0.82, and HEPES 15 (Barth’s solution at pH 8.2) or MES 15 (Barth’s solution at pH 5.5), with pH adjusted with NaOH. Inward proton currents were measured at a constant voltage of −20 mV, by switching the perfusing Barth’s solution from pH 8.2 to pH 5.5. Oocytes showing inward currents at pH 5.5 higher than 0.5 µA were used for macro-patch experiments.

Macro-patch pipettes were pulled from thin-walled borosilicate glass capillaries (1.7 mm O.D., 1.5 mm I.D., Harvard apparatus, Holliston, USA), coated with Sigmacote (Sigma-Aldrich, St. Louis, USA), fire-polished to a final resistance of 0.2 to 0.5 MΩ, and filled with Barth’s solution at pH 5.5. After removal of the vitelline membrane from oocytes in a hyperosmotic solution (ND96 solution at pH 8.2 plus 100 mM NaCl), oocytes were moved to the bath solution containing (mM) KCl 100, NaCl 5, MgCl_2_ 1, EGTA 1 and HEPES 15 (bath solution at pH 8.2 or 7.5) or MES 15 (bath solution at pH 5.5) with pH adjusted with KOH. After the macro-patch excision, currents were recorded at room temperature in the inside-out configuration under the perfusion of the bath solution. Recording of the currents with an Axopatch 200B (Molecular Devices) amplifier was done with a 21-fold repeated sequence of three episodes: A. holding potential at −20 mV for 100 ms. B. voltage step increasing in steps of 20 mV from −100 mV to +100 mV for 1000 ms, and C. tail potential of +20 mV for 460 ms. Data were low-pass filtered at 2 kHz and digitized with a sampling rate of 10 kHz. Current traces were analyzed with pCLAMP 10.2 (Molecular Devices) to generate IV curves.

## Results and Discussion

In order to study the influence of the pH on that side of the M2 protein, which is exposed to the interior of the viral particle, current-voltage relationships (IV curves) were measured in excised patches from *Xenopus* oocytes expressing the mutant M2D44A of the influenza A/Udorn/72 strain. This mutant was chosen for the experiments because it generates larger inward currents [20], [47] or outward currents [19] in *Xenopus* oocytes than the wild type channel. This increases the chance of measuring currents with little interference from endogenous currents. Also in a natural variant, the A/FP/Rostock/34 strain, Asp44 is replaced by the neutral Asn leading to an elevated conductance of this channel variant [19]. The reason for higher current of these mutants is supposed to be a greater instability of the Trp41-gate (as described in the Introduction) which yields a longer life time for the proton exchange between the His37-box and the viral interior. The analysis below shows that M2D44A is a suitable model for wt as the measured IV curves can be explained in terms of the structural data known from wild type M2.

From the pH dependency of the M2 protein and its mutants, which was found in previous studies mentioned above, we can assume that M2D44A in heterologous expression systems faces the external solution with the side that sees the endosome during the infection of cells. Hence, the C-terminal side, which faces the interior of the virus particle is on the cytosolic side of the oocyte in our expression system. It will be called “internal” or “cytosolic” side in the excised patches.

**Figure 2:**
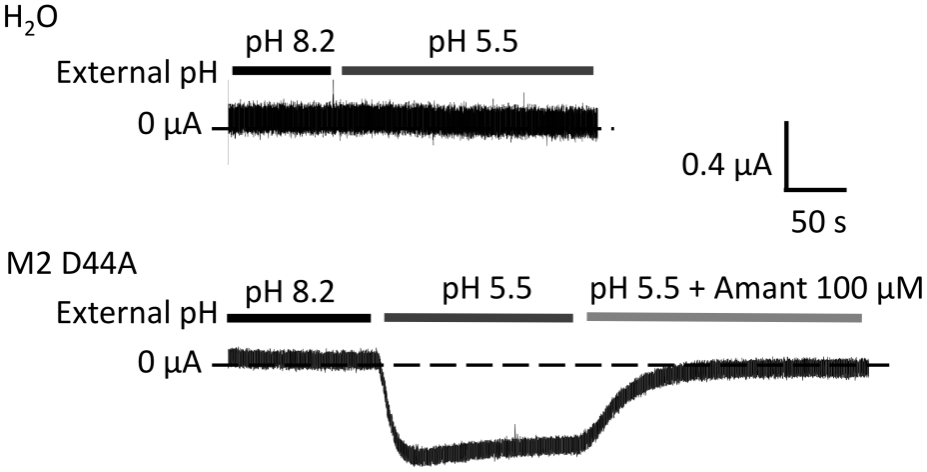
Activation of M2D44A by external acidification. Representative currents obtained from water- or M2D44A-injected *Xenopus* oocytes were recorded at −20 mV in TEVC. External pH was changed and amantadine added as a blocker as indicated by the bars. The internal pH was that of the oocyte, about pH 7.4.

First, we proved that our system reproduced the effect of external pH as already known from previous investigations [4], [39]–[41], [43]. Thus, TEVC experiments in oocytes injected with either water or cRNA of M2D44A were performed. Figure 2 shows a representative current trace at −20 mV of a water-injected oocyte. The current does not change when the bath pH is switched from pH 8.2 to pH 5.5. In contrast, the oocyte expressing M2D44A shows a change in current from 0.05 µA to −0.53 µA when the external pH is stepped from 8.2 to 5.5. This current can be blocked with the M2 blocker amantadine. The mutation M2D44A is known not to influence the amantadine block [20], [47].

### The effect of cytosolic pH_cyt_ on the IV curves of M2D44A

**Figure 3:**
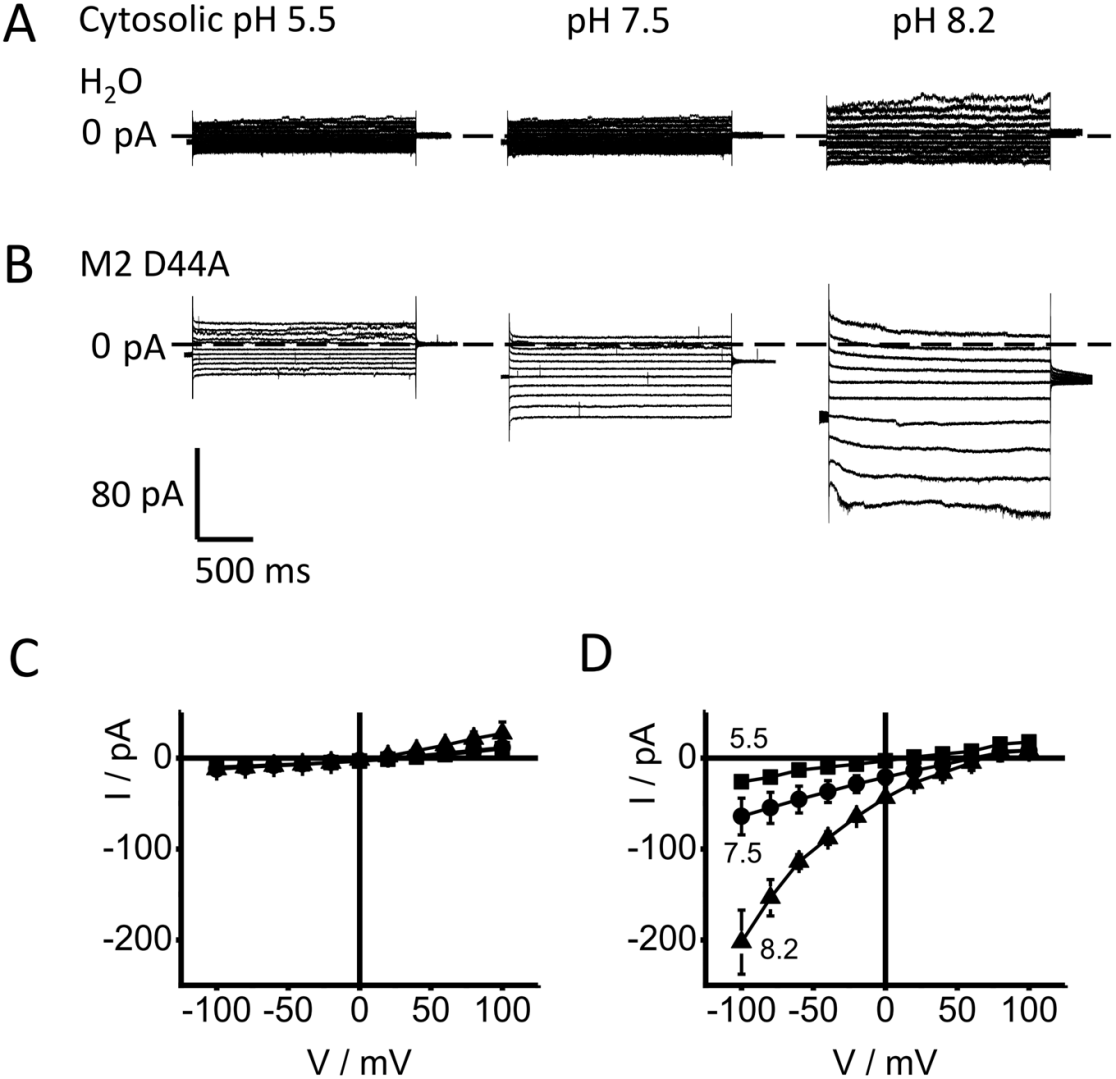
Dependence of currents on cytosolic pH in cell-free macro-patches. Representative traces are shown from (A) water-injected oocytes and (B) M2D44A-injected oocytes. The pH values are given at the traces. The averaged IV curves from patches clamped to test voltages between −100 mV to +100 mV are shown in (C) for H_2_O− and (D) for M2D44A-injected oocytes. The current traces at high negative membrane potentials (B) were noisy. However, the IV curves obtained after averaging show a smooth behavior at high negative potentials (D). The individual data is given in Table S1 in file S2.

To investigate the effect of cytosolic pH, current voltage relationships (IV curves) were measured on excised macro-patches. Representative recordings of membrane patches from a control oocyte and from an oocyte expressing M2D44A are shown in Fig. 3A, B. In these inside-out patches with an external pH of 5.5 in the pipette, the controls (Fig. 3A) exhibit only small currents which are only moderately affected by the cytosolic pH (pH_cyt_). In the presented example, only a small increase is found at a cytosolic pH of 8.2. The mean IV curves (Fig. 3C) from similar recordings confirm this trend.

In contrast, inside-out patches from oocytes expressing M2D44A exhibit a strong stimulation by alkaline pH_cyt_ (Fig. 3B, D). With pH_cyt_ = 5.5, the currents are small and not much larger than those from the controls. A shift to alkaline values causes a large increase in current being most apparent for the inward current. As expected for an H^+^ transporting channel, the reversal voltage shifts positive with an alkalinization of the cytosol. Below, it is shown that this shift is similar to that one calculated from the pH gradients after subtraction of the currents of the water-injected oocytes. A scrutiny of the IV curves of M2D44A at different pH_cyt_ discloses the following peculiarities:

1. The dependence on ligand concentration shows “transinhibition” [48], i.e., unidirectional outward current is hardly affected, but unidirectional inward current decreases with increasing cytosolic H^+^ concentration as revealed by the analysis below.
2. The IV curves do not saturate at high negative membrane potentials.
3. There is little outward current at positive potentials (inward rectification).

### Modeling of the IV curves

In order to draw conclusions about structure/function relationships in the M2 protein from the measured data, the IV curves in Fig. 3D were analyzed in quantitative terms on the basis of reaction kinetic models. In previous kinetic models, the different structural states related to the different protonation states of the His37-box (described in the Introduction) were accounted for by pH-dependent parallel transport models with transitions between them (e. g. [33], [45], [49]). Here, the basis for furnishing a kinetic model is the finding that the conformational changes of the Trp41-gate are in the same temporal range as the transition times of individual ions [15], [24], [32], [42]. This and the control of the Trp41-gate by the His37 protonation state disposed us to include the conformational changes as part of the transport cycle for protons through the pore. We start with a very simple model and learn from its failure how this model has to be augmented. At the end, a model comes out which can explain the peculiarities of the measured IV curves in terms of the structural findings described in the Introduction.

Common to all our models to explain the IV curves in Fig. 3D is the calculation of the measured current, *I*, as the difference between the absolute values of the unidirectional inward current and outward current



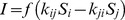
(1)with *S_i_* and *S_j_* being the occupation probabilities of adjacent states (specified below) in the chain of the reactions translocating the ion. *k_ij_* and *k_ji_* are the rate constants of the transitions between these states. The factor *f = e N P_O_* has to be set to an arbitrary value, because in the experiments in macro-patches, *N*, the number of channels, and *P_o_*, the open-probability remain unknown. Thus, all rate constants evaluated below contain an unknown factor related to the arbitrary choice of *f*. However, the ratios of the rate constants are independent from *f.*

### Two models of the effect of cytosolic pH, which fail, but highlight the necessity of the salient features of the successful model

The most prominent effect is “transinhibition” [48], i.e., the increase in inward current with decreasing cytosolic H^+^ concentration. At a first glance, it may be not surprising that the inward currents at pH 7.5 are smaller than those at pH 8.2 because the driving force (consisting of membrane potential plus the Nernst potential resulting from the difference in proton concentrations) is smaller at pH 7.5 than at pH 8.2. However, the relationship between driving force and flux is straightforward only around the reversal potential (zero current). Far away from the reversal potential, the unidirectional currents have to be considered. In Eq. 1, *S_ext_* = outward H^+^ concentration driving inward current is constant (pH_ext_ = 5.5) and independent on cytosolic pH. The outward flux, which depends on cytosolic pH is so small that it does not influence the measured current *I*, because the currents are measured far from the reversal potential. Thus, the IV curves for different pH_cyt_ are expected to coincide at negative potentials.

However, there is a strong difference between the IV curves in Fig. 3D. This so-called “transinhibition” or “inverse” effect of pH_cyt_ (effect on inward flow instead on outward flow, [48]) can already be generated by a simple binding site in the pore, as described by the reaction scheme of an enzyme.
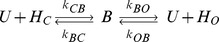
(2)


The reaction scheme of Eq. 2 is also the core of most of the kinetic models used to fit data from M2 (e.g. [42], [43], [45]). The cytosolic proton (*H_C_*) binds to *U* (state of the His37-box with two protons) leading to the bound form *B* (state of the His37-box with three protons). Dissociation can occur towards the cytosolic side via the rate constant *k_BC_* or to the outside (*O*) via *k_BO_*. According to Eq. 1, the H^+^ current conducted by the M2 protein is the sum of the (signed) unidirectional currents between two states e.g. between *U* at the cytosolic side and *B* at the His37 box or between *B* and outside *O*.
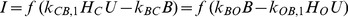
(3)with *H_C_* being the cytosolic, *H_O_* the outside H^+^ concentration. The rate constants *k_ij_* are defined as given at the arrows in Eq. 2. With only two indices, the unit of the rate constants is s^−1^. In bimolecular or voltage-dependent reactions, the *k_ij_* of Eqs. 1 or 3 include the influence of proton concentration and/or voltage. If the actual proton concentration is not included, they have an additional index “1”, e.g., *k_CB,1_* being *k_CB_* for 1 M *H_C_* (see Eq. 4a). If voltage is not included, the index is “0”, e.g., *k_BO, 0_* is the value of *k_BO_* at 0 mV (see Eq. 4b, c). For the sake of simplicity, the occupation probabilities of a state are given by the symbol of that state without brackets.

The dependence on voltage *V* and on cytosolic H^+^ concentration *H_C_* is incorporated as follows
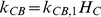
(4a)

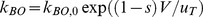
(4b)

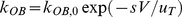
(4c)


**Figure 4:**
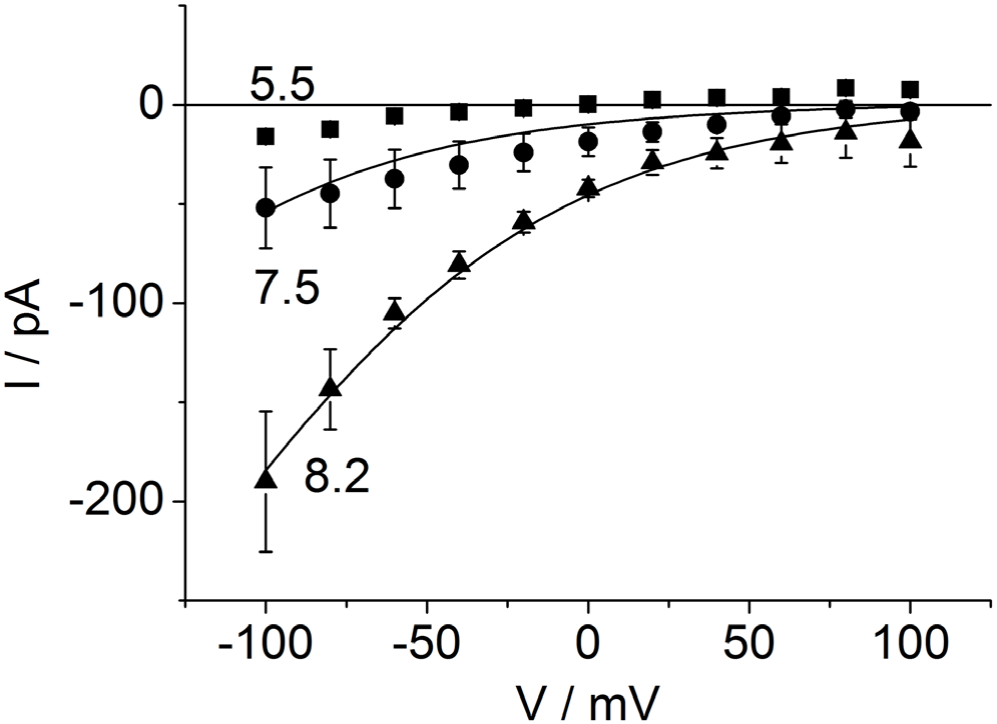
Averaged IV curves of M2D44A obtained from macro-patches at different cytosolic pH: 5.5 (squares), 7.5 (circles) and 8.2 (triangles) (n = 2, 3, 3, respectively). The error bars give the standard deviation of the measured data points. The external pH was 5.5. Averaged IV curves from water-injected oocytes (different pH values were measured on each control oocyte) have been subtracted from the original IV curves measured in patches expressing M2D44A (n = 5–7 per pH value) in order to isolate the M2 current from the endogenous currents. The data for pH_cyt_ = 7.5 and 8.2 was fitted with the linear model with a strongly binding residue (enzyme model) in the pore (Eqs. 2 and 3). Voltage acts on the *B-O* transitions (Eqs. 4b, c). Cytosolic H^+^ binds to the *U-B* transition *k_CB_* (Eq. 4a). Since the fit is not perfect, in particular for the data at pH 7.5, the full equations used for fitting are not given here, but in Eq. S4 in file S1).

Equation 4c uses the value of *k_OB, 0_* for pH_ext_ = 5.5. The electrical location of the Eyring barrier is presented by *s*, and *u_T_* = *RT*/*F* = 25 mV with *R*, *T, F* having their usual meaning. The model related to Eq. 2 is a primitive model. It can successfully fit the individual IV curve at pH 8.2, but fails in a global fit (Fig. 4). A global fit means that the same parameters are used for both values of pH_cyt_ = 8.2 and 7.5. (Note that *k_CB, 1_* and not *k_CB_* is a fit parameter according to Eq. 4a.).

Figure 4 is presented here because it highlights the salient features of all models tested here, namely transinhibition, inward rectification and exponential rise of current with negative potentials. Fixing the scaling factor *f* at 100, a global fit of the IV curves at pH 7.5 and 8.2 resulted in *k_CB_* = 2.5E13 *H_C_*, *k_BC_* = 4.6, *k_BO_* = 1.0E–3 exp (0.55 V/25 mV), *k_OB_* = 5.4E09 exp (–0.45 V/25 mV) for pH_ext_ = 5.5. As mentioned above, the values of the rate constants (given in s^−1^) are scaled by the unknown factor *f* = *e N P_O_.* Furthermore, the high value of *k_CB_* only has the meaning “very high”, because the fitting routine (also in the models below) becomes very insensitive to this value if it is high enough. The high value of *k_CB_* does not mean that many protons jump from *C* to *B*. Instead, it is the extremely high probability that the proton immediately jumps back after it has come from *B* to *C*. It seems to be well established that the binding site for protons is the His37-box [8], [12], [13], [22], [38].

However, the fit in Fig. 4 is poor. While the theoretical curve is still inside the error bars, it is obvious that the curvature of the theoretical curve at pH 7.5 is stronger than that of the experimental data. The next attempt to improve the fitting was to account for a putative role of Val27 as a constriction zone which controls the proton exchange between the His37-box and the external medium [14], [15], [26], [37], [50]. Thus, we have added a diffusion step to the enzyme, which leads to the following kinetic scheme
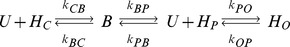
(5)


The index *P* labels a putative H^+^ pool in the cavity lined by Ala30, Ser31 and Gly34. However, the introduction of the putative pool results in a non-significant improvement of the fit, which is not distinguishable from the fit result in Fig. 4; thus the fit is not shown. The same poor fits were obtained regardless of whether the voltage-sensitive reactions were assumed between enzyme *B* and pool *P* or between pool *P* and external medium *O* or distributed to both steps (data not shown).

### A cyclic model, which describes the effects of the cytosolic pH of 7.5 and 8.2 correctly

**Figure 5:**
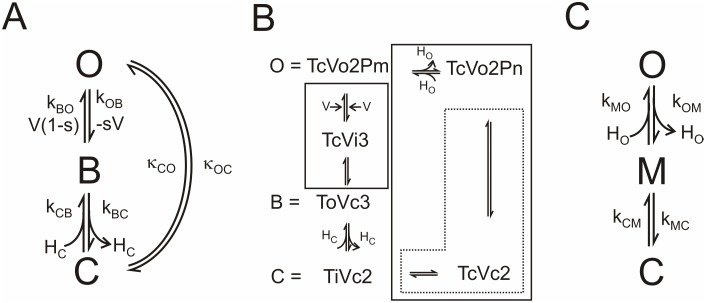
Reaction schemes for the dependence of proton transport in M2D44A on cytosolic pH. (A) Cyclic reaction with the following states of the M2 protein. *O*: the external part of the protein has taken up a proton from the external side or is ready to release a proton to the external solution. *B*: the protein has bound a proton from *O* via the voltage-sensitive translocation *k_OB_* or from *C (k_CB_)* and is ready for the voltage-sensitive translocation of this proton to *O* (*k_BO_*) or release to *C (k_BC_)*, *C*: the protein is ready to bind a cytosolic proton and transfer it to *B* with the rate constant *k_CB_* or to take a proton from *B* (*k_BC_*) and release it to the internal side. The recycling step *O-C* includes the resetting of the conformational changes of the protein necessary for the transport of the next proton. It also includes the exchange of a proton with the external solution. (B) Augmented model derived from the structural information as detailed in the Introduction. The meaning of the symbols is as follows: Capital letter: T = Trp41-basket; V = Val27-gate, P = pool in the cavity filled with m or n protons, m>n; small letter o = open, i = open, but instable c = closed; Number: Protonation state of the His37-box. The equality sign indicates which state of the augmented model is assigned to a state in the model in (A). The solid boxes include reactions, which are merged to gross reactions in (A) as explained in file S1. The dotted box indicates the gross reaction *k_MC_* and *k_CM_* used in the recycling branch of the model in (C). (C) The recycling pathway with the gross rate constants *κ_OC_* and *κ_CO_* of (A) is split up into two steps: *O-M* with external proton binding *k_MO_* (and release *k_OM_*), and recycling (*M-C*) with the gross rate constants *k_MC_* and *k_CM_* as indicated by the dotted box in (B).

More successful is a cyclic model of the kind proposed for electrogenic pumps [51], [52], cotransporters [48] and K^+^ channels [53], [54]. Interestingly, it also worked best for bacteriorhodopsin, another H^+^ transporting protein [55], [56]. Here, we assume a cyclic transport model with 3 states as given in Fig. 5A. The core of this model is identical to that of the models of Eqs. 2 and 5 with the following features:

1. A proton from the external side is translocated. The rate constants of translocation *k_BO_* and *k_OB_* are voltage-dependent (Eqs. 4b, c). Outside proton concentrations *H_O_* is not explicitly mentioned (included in *k_OB, 0_*) as it is constant (pH_ext_ = 5.5).
2. The proton is released to the internal (viral) side via *k_BC_*. The reverse reaction constant *k_CB_* is proportional to the internal proton concentration (Eqs. 4a, S15 in file S1).

The next feature is new:

1. The structural information described in the Introduction (e.g. [14], [15]) suggests that the protein undergoes conformational changes during ion transport. Thus, a recycling step is introduced as depicted in Fig. 5A: After releasing the proton at the cytosolic side (considering inward flow), the protein has to undergo a conformational change before it is ready to take up the next proton from the outside solution. This so-called “recycling” step *κ_CO_* is assumed to be independent from voltage and cytosolic proton concentration.

First, we show that this cyclic model is capable of fitting the IV curves in Fig. 3D in a global fit for pH_cyt_ = 7.5 and 8.2. Then, it is discussed how the states of the cyclic model in Fig. 5A correspond to the states of a more augmented model (Fig. 5B) which is suggested from the structural studies.

A difference to the linear models in Eqs. 2 and 5 is in the meaning of the symbols *C, B, O* in Fig. 5A (and *M* in Fig. 5C). Here, they present conformational states of the protein: *B* has the same meaning as in the enzyme model, namely, the probability of His37 being in a 3-fold protonated state. However, *C* means His37-box with two protons open to the cytosolic side and *O* means His37-box with two protons open to the outside. The law of mass conservation implies that *C+B+O* = 1 (Eq. S9d in file S1).

Again, the current is calculated by means of Eq. 3, and the dependencies on voltage *V* and on cytosolic H^+^ concentration *H_C_* are incorporated by means of Eqs. 4a–c. External H^+^ concentration, *H_O_*, is not explicitly shown, as it is constant during the experiments here (but see the analysis regarding pH_ext_ below based on Fig. 5C). Again, *s* is the electrical location of the Eyring barrier between *B* and *O* and *u_T_ = RT/F* = 25 mV. The occupation probabilities *C* of the unbound residue and *B* of the bound residue are calculated in the Supplementary Material from the reactions scheme in Fig. 5A leading to Eqs. S13–S15 in file S1 used for fitting the IV curves.

According to the model in Fig. 5A, the difference between the three IV curves in Fig. 3D should be caused only by the influence of the proton concentration *H_C_* on the binding reaction (i.e, *k_CB_ = k_CB,1_ H_C_,* Eqs. 4a and S15 in file S1). To test this, the cytosolic H^+^ concentrations were set to different fixed values, namely *H_C_* = 3160 nM for pH 5.5, 31.6 nM for pH 7.5 and 6.3 nM for 8.2 as given in Table 1. All other parameters were free, but “shared” in the global fit, i.e., free but identical values of *s*, *k_CB, 1_, k_BC_, k_BO, 0_, k_OB, 0_, κ_CO_* (Fig. 5A) were used for the three different cytosolic pH values. *κ_OC_* was not a free parameter, but calculated by means of the law of microreversibility (Eq. S17 in file S1). The scaling factor *f* = *e N P_O_* (Eqs. 1 and 3) was set to an arbitrary value of 100 because of the unknown number of channels, *N*, as mentioned above.

**Table 1:** Parameters of the fits shown in Fig. 6.

		pH 8.2 global	pH 7.5 global	pH 5.5 global	pH 5.5 free	pH 5.5 free
Color in Fig. 6		red	red	red	black	black
*f*	pC	100	100	100	100	100
s*		*0.45*	*0.45*	*0.45*	0.45	0.45
*u_T_*	mV	25	25	25	25	25
*H_C_*	M	**6.3E–9**	**3.16E–8**	**3.16E–6**	**3.16E–6**	**3.16E–6**
k_CB,1_*	1/(sM)	*7.05E15*	*7.05E15*	*7.05E15*	7.05E15	**4.11E4**
*k_CB,1_ H_C_*	1/s	**4.46E7**	**2.23E8**	**2.23E10**	**2.23E10**	**0.13**
k_BC_*	1/s	*16092*	*16092*	*16092*	16092	**7.8**
k_BO,0_*	1/s	*0.0028*	*0.0028*	*0.0028*	**3.21**	**1.92**
k_OB,0_*	1/s	*0.72*	*0.72*	*0.72*	**0.028**	**0.27**
*κ_OC_*	1/s	2.7	2.7	2.7	**0.34**	**1.22E5**
κ_CO_*	1/s	*14500*	*14500*	*14500*	**1.86E7**	14500
*E_rev_, fit*	mV	156	115	0	0	0
*E_rev_, Nernst*	mV	159	118	0	0	0

The asterisks denote free parameters which were equal for the global fits at cytosolic pH 8.2, 7.5 and 5.5 (italic numbers). The bold numbers indicate values, which are different for different pH values. There are two “free” fits at pH 5.5. Here, only the non-bold, non-italic parameters were equal to those of the global fit for pH 7.5 and 8.2. Bold numbers are different from those of the global fit. “*E_rev_*, fit” is obtained from the intersection of the fitted curves with the voltage axis, “*E_rev_*, Nernst” is calculated from the pH gradient (pH_ext_ = 5.5).

**Figure 6:**
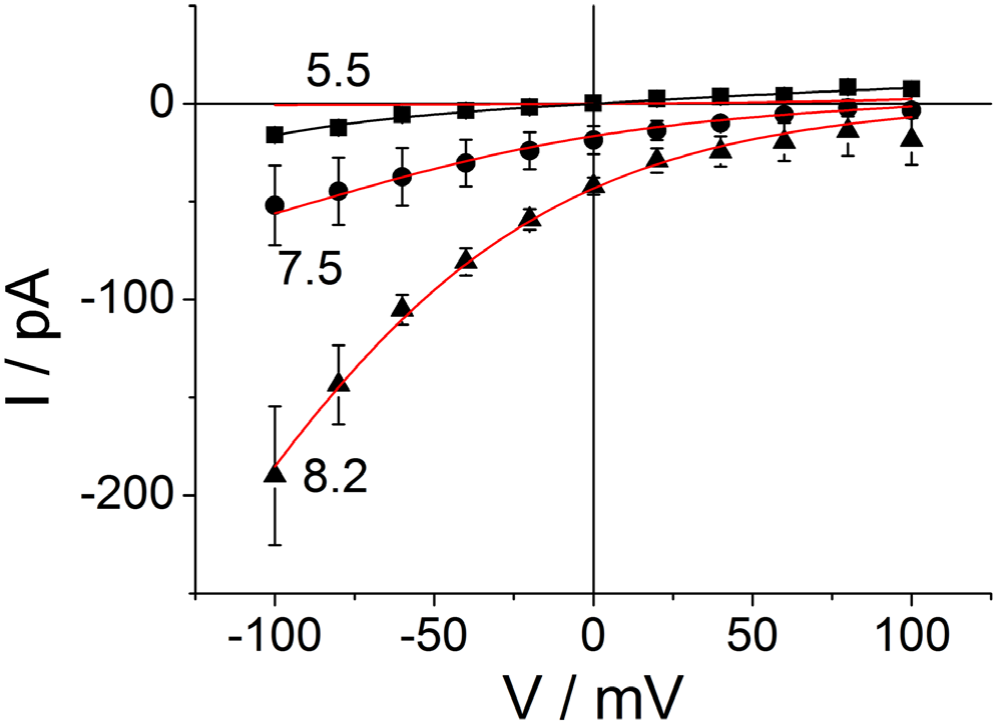
Fit of the data of Fig. 4 with the cyclic reaction scheme. The lines give the fits by means of Eqs. S13–S15 in file S1. For pH 5.5, two fits are shown: red curve: joint fit with curves for cytosolic pH of 7.5 and 8.2, black curve: without the restriction by the joint fit. This is illustrated by two different “free” fits at pH 5.5 (Table 1, data columns 4 and 5), which show that different sets of rate constants can fit the curve.

The results of the fits are shown as smooth lines in Fig. 6; the fitting yields a good coincidence between the calculated IV curves and the measured data for pH_cyt_ = 7.5 and 8.2. For pH_cyt_ = 5.5, two different curves (black and red) are shown. They will be discussed below.

The choice of an arbitrary value of *f* = *e N P_O_* implies that the absolute values of the rate constants in Table 1 remain uncertain as long as *f* is unknown. It is difficult to overcome this problem because the apparent low unitary conductance of the M2 protein prevents single-channel measurements, and *N*, the number of channels in the macro-patch, is unknown. In all our current recordings of M2D44A (similar to those in Fig. 3B), no gating and no excess noise (noise higher than baseline noise) was found also indicating that single-channel current of the M2D44A protein cannot be resolved. Consequently, only the ratios of the rate constants can be determined from the shape of the IV curves. There are reports on single-channel conductances. However, they range from 10 to 1000 protons per second [32], [41], [57] or 35 aS (estimated from pH changes, [58]) to 6 pS in 1 mM HCl [59]. Again, it has to be mentioned that the high value of *k_CB_* gives only the message “very high”, because the fitting routine becomes insensitive to *k_CB_* at high values.

The successful global fit of the IV curves at pH_cyt_ of 7.5 and 8.2 suggests that there is no pH-dependency of the rate constants besides the obvious linear effect of cytosolic pH on *k_CB_* in Eq. 4a. Thus, there is also no need to assume pH-dependent gating in this range of cytosolic pH (besides the conformational changes included in the model of Fig. 5B as discussed below). It is not surprising that the reversal potentials obtained from the extrapolation of the fitted curves in Fig. 6 (*E_rev_*, fit in Table 1) and those calculated from the proton gradients (*E_rev_,* Nernst) are quite equal as microreversibility (Eq. S17 in file S1) was introduced for the rate constants at pH_cyt_ = 5.5 = pH_ext_. Thus, the message is not based on the fact that these numbers are equal, but on the finding that these numbers yield good fits of the curves (if the scatter at pH 8.2 at positive potentials is ignored, and the extrapolation is taken as the true curve). This verifies that H^+^ is the dominating transported ion, at least at pH_cyt_ 8.2 and 7.5. At pH_cyt_ 5.5, this statement is much weaker as also the reversal potentials of K^+^ and Cl^−^ are close to zero.

### Relationship of the cyclic model in Fig. 5A to the actual knowledge of the conduction mechanism

Figure 5B shows the states of the conduction mechanism in M2, which can be extracted from the structural findings reported in the Introduction, especially in the work of [15]. The states are labeled as follows: The capital letters name the gate (V = Val27-gate, T = Trp41-basket), the small letters the state of the related gate (o = open, i = open, but instable, c = closed) and the number refers to the protonation state of the His37-box. In addition, two states give also the protonation state of the hypothetical “Pool” in the cavity at Ala30, Ser31 and Gly34 (P is omitted in the labels for the other states where it has no influence.).

Proton uptake occurs as follows: We start with the protonation state 2 of the His37-box. In state TcVo2, the Trp41-gate is closed and the Val27-gate is open and ready to bind an external proton. The distinction between TcVo2Pn and TcVo2Pm (m>n) is not crucial for the model, and the two states may be merged. It offers the possibility to include that the cavity at Ala30, Ser31 and Gly34 is a pool *P* (see Eq. 5) which can store some protons. Voltage draws the proton from the pool towards the His37-box. When the proton has bound to the His37-box (thus increasing its protonation state from 2 to 3) the Val27-gate starts to close. The introduction of the state TcVi3 accounts for the possibility that protonation-induced tilting of the helices [15], [33] takes some time (milli- to microseconds). Thus, the Val27-gate is in an instable state for a short time, before it is converted to the state ToVc3. Stochastic transitions from ToVc3 to TcVi3 are necessary to enable outward flow. When the conformational change has stabilized after the transition from TcVi3 to the state ToVc3, the Trp41-gate is open. Now, the His37-box can lose the third proton to the cytosolic side, thus reaching the state TiVc2. Again, we assume that the tilting of the helices takes some time, and the Trp41-gate is transiently open for exchange between His37-box and viral interior. After a short time in TiVc2, the Trp41-gate closes resulting in state TcVc2. Statistical fluctuations leading to the inverse transition TcVc2 to TiVc2 have to be assumed to enable outward current. The next step is the opening of the Val27-gate in state TcVo2, and the cycle can start again. The return to the state TcVo2 is the recycling step. Zhou [49] also considers the Val27-gate. However, in that approach the gating is stochastic, not related to His protonation. Thus, it does not introduce a recycling step, but just a scaling factor for the rate constants *k_OB_* and *k_BO_*.

For fitting the IV curves, the states in Fig. 5B are presented by the cyclic 3-state model in Fig. 5A. This is done by merging adjacent reactions into gross reactions as indicated by the solid boxes in Fig. 5B. The theoretical background [52], [60] and the implications and caveats of this procedure are described in file S1.

It depends on the relative stabilities of the Val27-gate and of the Trp41-gate, whether the intermediate state TcVc2 occurs or whether closing of the Trp41-gate and opening of the Val27-gate occur simultaneously. This has no influence on fitting the data, since these states in Fig. 5B are merged into the gross rate constants *κ_OC_* and *κ_CO_* of Fig. 5A. The increased opening of the Trp41-gate [19] as e.g. found in the M2D44A mutant could be a longer dwell-time in state TiVc2, which can be brought about by a modification of the rate constants between TiVc2 and TcVc2.

Even though the approach is different, the model in Fig. 5A is kinetically similar to the models of Zhou [49] and Polishchuk et al. [33]. In those models, transport occurs in two parallel cyclic models, model B stands for the conformational state found at intermediate pH and model C for low pH. In each submodel, protons can bind and dissociate leading to transitions between BH2 and BH3 or CH2 and CH3 (here B and C are not identical with *B* and *C* in Fig. 5A). In addition, there are two parallel reactions called leaks. Furthermore, there are transitions BH2/CH2 and BH3/CH3. The leaks were found to carry about zero current. Then, the pathway BH2-BH3-CH3 corresponds to the *O-B-C* path in Fig. 5A and CH2/BH2 is the recycling path. Unfortunately, we cannot compare the rate constants because of different experimental conditions.

A final remark deals with the mixing of gating and binding in the models of Fig. 5 which may support the assignment of M2 to the class of transporters, as suggested e.g. by [32], even though this distinction may be a matter of debate. The similarity between M2 and a transporter like the lactose permease [61] includes even the transport-related tilting of the helices. Kinetically, the transporter model corresponds to the ancient carrier models, which still apply to mobile carriers like valinomycin [62]. “ToVc” corresponds to “accessible for the ligand from the inside”, and TcVo to “accessible for the ligand from the outside”. In the case of previous M2 modelling, the expressions “accessible” or “inaccessible” have already been used by Pielak and Chou [42].

### The peculiarities of cytosolic pH of 5.5

The red curve for pH_cyt_ = 5.5 in Fig. 6 shows that the IV curve for pH_cyt_ = 5.5 cannot be fitted in a global fit together with pH_cyt_ = 7.5 and 8.2, because in a global fit the currents for pH_cyt_ = 5.5 become by far too small as indicated by the red curve coinciding with the voltage axis.

The failure of a global fit including data with pH_cyt_ of 5.5 can be explained by one or both of the following effects.

1. Cytosolic pH modifies the kinetics of the protein.
2. Other ions besides H^+^ are transported.

In the case of the first hypothesis, pH_cyt_ of 5.5 would modify the kinetic properties of the protein. However, a single IV curve without the constraints from a global fit does not have enough characteristics to provide a unique determination of the involved rate constants. For instance, data column 4 in Table 1 shows that the values of *k_CB_* and *k_BC_* of the global fit can be maintained if the other three rate constants are free. In the fit of data column 5, *κ_CO_* was taken from the global fit. Then, the other four parameters had to be free, etc. Nevertheless, the fits show that a regulatory (allosteric) effect of pH_cyt_ of 5.5 cannot be excluded.

In the case of the second hypothesis, several authors found evidence that the M2 protein does not only transport H^+^, but also other ions which are necessary for charge balance during infection, e.g. K^+^. Even though the ratio of permeabilities of H^+^ to monovalent cations was estimated to be around 10^6^:1 [43], [44], K^+^ or Na^+^ can contribute to the current because of their much higher concentration. Even Cl^−^ may be involved as suggested from the role of Cl^−^ found in the MD simulations of Wei and Pohorille [38]. Remarkably, also in the H^+^ transporting bacteriorhodopsin, a conductivity for other ions had to be assumed for a good fit of the IV data [55]. At low H^+^ currents as found with pH_cyt_ = 5.5, the currents of other ions may become dominating. In that case, the fit results in the data columns 4 and 5 of Table 1 are only suggestions for the rate constants of this second pathway. Their exact values need to be determined in additional experiments in a system without disturbing endogenous currents (e.g. bilayers).

### Conclusions drawn from the fits in Figs. 4 and 6

**Figure 7:**
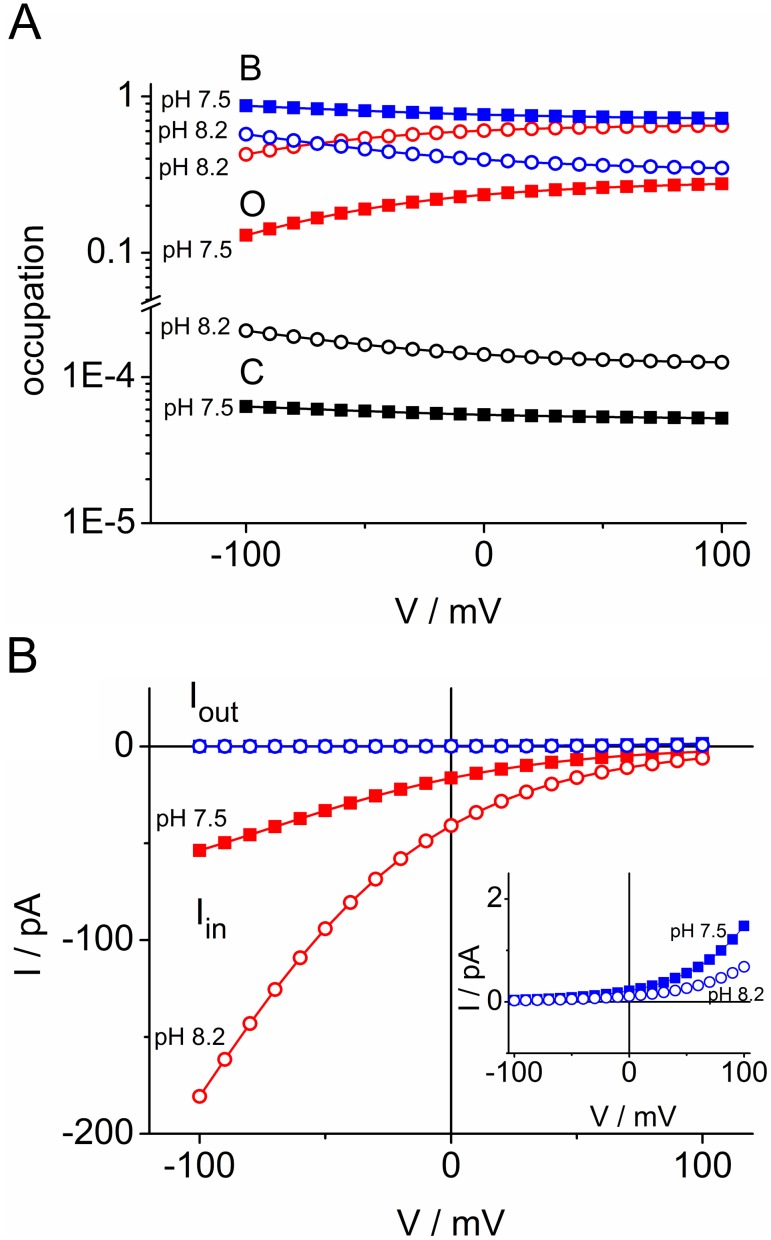
Properties of the M2D44A transport cycle as calculated from the cyclic model in Fig. 5A. (A) Occupation probabilities of the three states in the model and their dependence on membrane potential *V* and cytosolic pH_cyt_. (B) Unidirectional currents *I_in_ = −f O k_OB_* (red) and *I_out_* = *f B k_BO_* (blue, pH 7.5 and 8.2 coincide) and their dependence of membrane potential *V* and cytosolic pH_cyt_. The inset shows the unidirectional outward currents on an expanded scale.

The mechanisms behind the characteristics of the IV curves in Fig. 6 become obvious from a calculation of the occupation probabilities of *C, B* and *O* (Fig. 7A) and the unidirectional currents between states *B* and *O* as calculated from Eqs. S10 and S11 in file S1 for the cyclic model of Fig. 5A (Fig. 7B). The occupation probability of the state *B* (His37-box protonated by 3 protons) determines the outward current *I_out_ = B k_BO_.* Since this current is close to zero (Fig. 7B), it does not matter that *B* is somewhat smaller at pH_cyt_ = 8.2 than at pH_cyt_ = 7.5. The rate constant *k_OB_* is much greater than *k_BO_*. Thus, the inward current *I_in_ = –O k_OB_* is about equal to the net current *I*. Since the occupation of state *O* is strongly dependent on pH_cyt_ (Fig. 7A), the inverse effect (transinhibition) is generated: Inward current decreases and not outward current increases with increasing cytosolic H^+^ concentrations. This is an effect of the high affinity of *B* making *k_OB_* greater than *k_BO_* and making the two-fold protonated state *O* very sensitive to pH_cyt_.

In Fig. 7B, inward and outward currents were calculated from the fluxes between *O* and *B.* In the case of *B–C,* such a graph would not tell very much because the absolute values of both fluxes would coincide (data not shown). They are about equal to *f B k_BC_*, i.e. a little bit lower than 1600000 pA (*f k_BC_* in Table 1), due to the small deviation of *B* from 1. However, calculating the difference between the absolute values of *I_in_* and *I_out_,* yields values of 0 to 200 pA resulting in the (expected) coincidence with *I_in_* = *I* shown in Fig. 7B. This results from a slightly stronger relative variation of *C* than of *B* with pH_cyt_ and voltage. The message is as follows. From 1600000 pA flowing from *B* to *C* (in the macro-patch, not in a single protein) during the short open time of the Trp41-basket, all flow back, except a minority of 0 to 200 pA which escape to the cytosolic side.

The exponential increase of inward currents in Fig. 6 results from the only moderate decrease of *O* with negative membrane potentials (Fig. 7A), which is too weak to compensate the exponential increase of *k_OB_* with voltage (Eq. 4c). Thus, saturation is not observed in the window of accessible potentials. Alternatively, the exponential increase can be explained by a general rule for cyclic models [48], namely that the voltage-insensitive reactions of a transport cycle comprising *k_BC_* and *κ_CO_* are faster than the voltage-dependent *k_OB_* (Table 1). The strong inward rectification results from the feature that *k_BO, 0_* and *κ_OC_* are small as compared to *k_OB, 0_* and *κ_CO_* (Table 1).

The recycling step in Fig. 5A, related to the gross rate constants *κ_CO_* and *κ_OC_,* turned out to be necessary for the good fits in Fig. 6. The same was found in bacteriorhodopsin [55], [56]. In bacteriorhodopsin or sensory rhodopsin, the movement of the proton alters the charge distribution in the protein and thus influences the tertiary structure. These structural changes have to be reset before a new proton can be transported [63]–[65]. In the case of M2, it is also protonation, which is responsible for structural changes during transport as reported in the Introduction and in the description of Fig. 5B.

### Effects of external pH

The effects of external pH on H^+^ flux through M2 have been investigated [4], [39]–[41] and modeled in previous papers [33], [42], [43]. A bulk of published data shows that M2 exhibits a strong decrease of inward currents with external alkalinization, which also becomes obvious in Fig. 2. Even though the external pH was not in the focus of the present study, we test under which conditions the cyclic model of Fig. 5A predicts these findings.

Since the effect of pH_ext_ is not explicitly introduced in the cyclic model of Fig. 5A, we have to inflate the 3-state model to a 4-state model. For this issue, we partially decompose the recycling reactions *κ_OC_* and *κ_CO_* of Fig. 5A into two reaction steps. Now the state TcVo2Pn is called *M*, and TcVc2 is included in the gross reactions *k_CM_* and *k_MC_* as indicted by the dotted box in Fig. 5B. Thus, we have a pH_ext_-sensitive reaction *k_MO_* between *M* and *O*. The other rate constants *k_OM_*, *k_MC_*, *k_CM_* (Fig. 5C) are constant. Fortunately, the complex equation of the resulting 4-state model is not needed. Inward current was found to increase with increasing external proton concentrations [41] as expected from the change in driving force. Thus, there is no transinhibition involved, and this finding is used in file S1 to conclude that here the rate constant of proton binding (*k_MO,0_ H_O_*) is much smaller than that of the recycling step *k_MC_* in Fig. 5C. Under this favorite condition, the effect of pH_ext_ can be incorporated into the 3-state model of Fig. 5A via the 3-state recycling reactions *κ_OC_* and *κ_CO_* (Eq. S18a, b in file S1), i.e., *κ_OC_* remains unchanged and
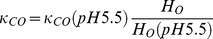
(6)


**Figure 8:**
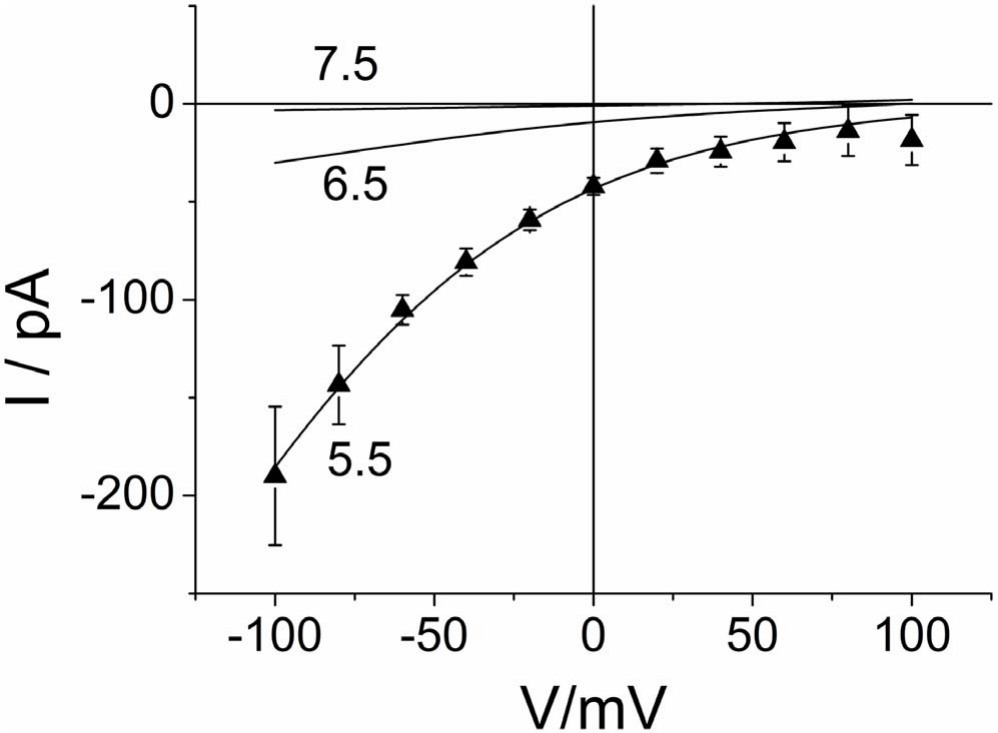
Calculated IV curves for different external pH of 5.5, 6.5 and 7.5. The parameters in Table 1 from the joint fit for pH_cyt_ = 8.2 with *κ_CO_* modified by pH_ext_ (Eq. 6) were used to generate the curves; pH_ext_ is given at the curves. For comparison, triangles show the experimental data of Fig. 6 for pH_cyt_ = 8.2/pH_ext_ = 5.5.

The curves in Fig. 8 are obtained with the rate constants of Fig. 6 (or Table 1, data column 1, pH_cyt_ 8.2) with *κ_CO_* modified according to Eq. 6.

In terms of the models in Fig. 5A, the decrease of inward current with a decrease in outside H^+^ concentration results mainly from a decrease of the occupation probability of state *O* = TcVo2Pm, thus determining the uni-directional flux *I_in_* = *−f O k_OB,0_ exp (−sV/u_T_).* In a less augmented model, the state TcVo2Pm of Fig. 5B might be absent, and proton binding coincides with voltage-driven translocation. This would lead to *I_in_* = *−f O H k_OB,0_ exp (−sV/u_T_)* with *O* now being state TcVo2 (then the label Pn is no longer necessary). In any case, a major effect of the open-closed transitions of Val27 (besides those in Fig. 5B) is not involved in our data.

This statement does not contradict conclusions from recent measurements of proton conduction rate [42], in which mutation of Val27 to a smaller hydrophobic residue has yielded an increased proton flux. The significance of Val27 as a gate for proton transport through the channel as recognized in MD simulations [37], [66] may have a more complicated phenomenology, which may turn up under different experimental conditions. For explaining the measured data here, it is sufficient to account for the coupling between His37 protonation and Val27 closure as done in Fig. 5B.

## Conclusions

The present data show that the activity of the D44A mutant of the M2 proton channel can be measured in excised membrane patches. This allows a direct control over the pH on that side of the channel, which is normally exposed towards the interior of the virus particle. It turned out that the IV curves measured in M2D44A can be modeled in agreement with the structural data obtained from wild type M2. Due to the higher open probability of the Trp41-gate of M2D44A, it is expected that the rate constants related to ToVc3, TiVc2 and TcVc2 may have different values in wild type. It remains open whether the model in Fig. 5B comprises too much or not enough (most likely) states. For our data, this does not matter because of the reduction of the augmented model in Fig. 5B to a 3-state model in Fig. 5A. The global fitting of the IV curves measured at pH_cyt_ 7.5 and 8.2 leads to the following messages for the function of the M2D44A channel: 1. There is a strong binding site at the cytosolic side, which is assigned to the His37-box. This strong binding is the origin of the effect of transinhibition, i.e., the decrease of inward current with increasing cytosolic proton concentration. 2. A cyclic reaction scheme is necessary to obtain a good global fit. This implies that the protein undergoes conformational changes during a transport cycle. They are assigned to the effect of protonation of the His37-box on the Trp41-basket and Val27-gate as revealed by structural analysis [14], [15], [19]. These changes have to be reset before the protein opens for the uptake of the next proton. 3. The IV curves rise exponentially with hyperpolarizing membrane potential. This implies that the voltage-sensitive step is the slowest one in the transport cycle.

The current measured at cytosolic pH 5.5 is too large to be described by the model for pH 7.5 and pH 8.2. Experiments in a system with less endogenous currents (e.g. bilayers) have to be done in order to distinguish between two different scenarios, namely regulation (i.e., modification of the involved rate constants by acidic cytosolic pH) or involvement of other ions also transported by the M2 protein.

## Supporting Information

File S1 Model calculations.
(DOCX)

File S2 Table S1. Averaged IV curves from macro-patches from water-injected control oocytes and oocytes injected with M2D44A RNA.
(XLSX)
