# Supplementary material for: Effect of Cytosolic pH on Inward Currents Reveals Structural Characteristics of the Proton Transport Cycle in the Influenza A Protein M2 in Cell-Free Membrane Patches of Xenopus oocytes
Source: PLoS One. 2014 Sep 11;9(9):e107406. doi: 10.1371/journal.pone.0107406 (PMC4174909; doi:10.1371/journal.pone.0107406)
Supplement: File S1 — Model calculations. (DOCX) [file pone.0107406.s001.docx]

**Supplementary Information**

**Model 1: The "enzyme" model**

The enzyme model (Eq. 2) assumes a binding site in the pore of M2 with *B* being the residue having bound the third proton, which will be translocated, and *U* (unbound) this residue without this proton. Setting up the rate equation from the reaction scheme of Eq. 2 for the bound residue *B*

( S1 )

and incorporating mass conservation

( S2 )

leads to the steady state occupation probability of *B*

( S3 )

Nomenclature: Rate constants of the form *k_IJ_* have the unit s^-1^. In bimolecular or voltage-dependent reactions, the *k_IJ_* include the influence of proton concentration and/or voltage. If the actual proton concentration is not included, they have an additional index “1”, e.g., *k_CB,1_* being *k_CB_* for 1 Mol H^+^. If voltage is not included, the index is “0”, e.g., *k_BO,0_* being the value of *k_BO_* at 0 mV. For the sake of simplicity, the occupation probabilities of a state are given by the symbol of that state.

The measured current is the difference between the absolute values of the uni-directional currents between two adjacent states, as given by Eq. 3.

Inserting *B* (Eq. S3) and the dependencies of the rate constants on voltage *V* and on proton concentration *H_C_* (Eqs. 4a-c) into Eq. 3 leads to the equation used for fitting the IV curves

( S4 )

with *e* being the charge of the proton, *N* the number of M2D44A proteins in the patch and *P_O_* the open-probability (which is not included in the reaction kinetic equations here).

**Model 2: The enzyme model with constriction zone at the outside**

The reaction scheme for this scenario is given by Eq. 5, which leads to the following equations

( S5a )

( S5b )

( S5c )

The steady-state solutions of Eqs. S5a to c plus mass conservation equal to that of Eq. S2 yield the occupation probability of *U*

( S6 )

This equation leads to a numerical problem with Origin®. The square root and the term before the square root differ by such a small relative amount that the second term in the square root is ignored. On the other hand, the small value of this term makes it legitimate to use the approximation $\sqrt{1+a}=1+\frac{a}{2}$ leading to

( S7 )

Then, the current can be calculated as follows (*f = e N P_O_*)

( S8a )

( S8b )

**Model 3: The cyclic model**

In the cyclic model (Fig. 5A) a recycling step is introduced. Actually, this kind of model has arisen from the concept of carrier-mediated transport [1] and was further developed by Hansen et al. [2],[3],[4]. The recycling step represents the return of the carrier, e.g. valinomycin, after it has delivered its ligand. However, the model has turned out to apply also for immobile ion transporters such as electrogenic pumps, channels or cotransporters as mentioned in the main text. In those objects, the recycling of the carrier seems to be replaced by the resetting of the conformation of the protein, which has been modified during the transfer of an ion.

The model in Fig. 5A leads to the following rate equations

( S9a )

( S9b )

( S9c )

( S9d )

Here, *C*, *B* and *O* label conformational states of the protein as explained in the main text. The same symbols are also used for the occupation probabilities of these states (or relative times spent in the related states in single-channel experiments) or the relative number of M2D44A residing in that state in whole-cell or macro-patch studies. The sum over all conformational states of the protein is 1 (Eq. S9d). The zeros at the right-hand side of the first three equations imply that the IV curves are measured under steady-state conditions. Calculating the occupation probabilities *C*, *O* and *B* from Eqs. S9a to d results in

( S10a )

( S10b )

( S10c )

The unidirectional currents between *O* and *B* are

( S11a )

( S11b )

The net current transported by a single M2D44A in the open-state is the difference between the absolute values of the unidirectional fluxes, which can be calculated from each pair of states. Here we use the voltage-dependent unidirectional fluxes between *B* and *O* (Eq. S11a,b)

( S12 )

The insertion of *B* and *O* leads to

( S13 )

with *e* being the charge of the proton, *N* the number of M2 proteins in a macro-patch and *P_O_* the open-probability

The voltage dependence is introduced by the assumption that the voltage-sensitive reactions *k_BO_* and *k_OB_* have to overcome an Eyring barrier at the electrical distance *s* from the external side and (1-*s*) from the cytosolic side.

( S14a )

( S14b )

Binding of protons at the cytosolic side influences *k_CB_* (Eq. 4a)

( S15 )

This leads to the full equation

( S16 )

For fitting, Eq. S16 was not used. Instead, Eqs. S13 to S15 were implemented in Origin® for fitting the IV curves measured at cytosolic pH_cyt_ of 5.5, 7.5 and 8.2 (with external pH_ext_ = 5.5).

Furthermore, it is assumed that M2D44A is not fueled by ATP. Thus, the condition "zero current at zero voltage at equal pH on either side" is implemented by the equation of microreversibility

( S17 )

with *k_CB,pH5.5_* being *k_CB_* at pH_cyt_ = pH_ext_ = 5.5. This means that the clockwise product of the rate constants is equal to the counter clockwise product if pH_cyt_ = pH_ext_.

**Implementing external proton binding into the 3-state model**

Fig. 5C shows the extension of the 3-state model (Fig. 5A) by a 4-state model. There, the *O-M-C* reaction scheme of Fig. 5C should replace the *O-C* recycling step (*κ_OC_* and *κ_CO_* in Fig. 5A). Proton binding is related to *k_MO_* and proton release from *O* into the outer phase to *k_OM_*. Then, *κ_OC_* and *κ_CO_* can be expressed in terms of the 4-state branch in Fig. 5B (see next paragraph: The use of minimum models).

( S18a )

( S18b )

The second part of Eqs. S18a,b holds if *k_MC_* >> *k_MO,1_ H_o_*. In th­at case, *κ_CO_* becomes proportional to the external proton concentration *H_o_*

( S19 )

It may be argued that now *M* is missing in Eq. S9d (constant sum of states).

However, the probability of being in state *M* is independent of *H_o_* (pH_ext_) under the numerical situation (*k_MC_* >> *k_MO,1_ H_o_*) employed here:

( S20 )

This implies that the constant value of *M* can be included by constant reserve factors (which are unknown anyway) in *O* and *C*. Thus, *M* has no effect on the product of *f* = *eNP_O_* and the individual rate constants. Consequently, the scaling factor *f* remains the same for all values of pH_ext_, and Eq. S19 can be used to understand the current inhibition by high external pH values.

**The use of minimum models**

Reactions in proteins can comprise a plethora of states and transitions between them, and many of them do not become obvious in a given experiment. Nevertheless, kinetic reaction schemes with a smaller number of states are often employed and even used for fitting. This is legitimate and can provide valuable insights, if the caveats of this approach are kept in mind. From the structural investigations discussed in the Introduction, the states shown in Fig. 5B can be extracted. However, the IV curves in Fig. 3D provide enough information only for the cyclic 3-state model in Fig. 5A. There may be even more undetected states, but that does not make a difference as becomes obvious from the following considerations.

Merging the model with the states in Fig. 5B into the cyclic 3-state model in Fig. 5A. is done by introducing gross rate constants as indicated by the boxes in Fig. 5B. In order to show the principle let us assume a reaction

( S21 )

The intermediate state *J* is

( S22 )

The sum of states is

( S23 )

with *r_KJ_* and *r_HJ_* being defined by the comparison of the middle and right-hand terms in Eq. S22.

The factors *r_H_* = 1 + *r_HJ_* and *r_K_* = 1+ *r_KJ_* are called reserve factors [2],[3]. (Do not use the inverse definition of [1]). The gross rate constants are

( S24a,b )

The flow between H and K has to be same in the original and in the merged model

( S25 )

The same holds for the inverse flow. The occupation probabilities and gross rate constants labeled by “app” = “apparent” are those, which are revealed by the analysis of a reduced model, i.e. by fitting with the model in Fig. 5A. These apparent state occupations of *H* and *K* are greater than the original ones, and the gross rate constants are smaller due to the reserve factors (e.g. *r_H_* = 1 + *r_HJ_*) . The problem is that these reserve factors are unkown as long as the reactions between *J* and its neighbors remain unkown. However, there is no way out as long as the intermediate reactions are not tackled by the experimental conditions or background information is not available. (This obviously is the reason that this problem is rarely mentioned in a kinetic analysis). Nevertheless, the analysis in spite of unknown intermediates is done frequently, because the apparent occupation probabilities and gross rate constants provide a first estimation especially when the occupations of the hidden intermediate states are small thus leading to small reserve factors (Eq. S22). Furthermore, the results can be transformed when the model is expanded in new experiments [4].

**References**

1. Stark G, Ketterer B, Benz R, Läuger P (1971) The rate constants of valinomycin-mediated ion transport through thin lipid membranes. Biophys J 11: 981–994.

2. Hansen U-P, Gradmann D, Sanders D, Slayman CL (1981) Interpretation of current-voltage relationships for “active” ion transport systems: I. Steady-state reaction-kinetic analysis of class-I mechanisms. J Membr Biol 63: 165–190.

3. Hansen U-P, Tittor J, Gradmann D (1983) Interpretation of current-voltage relationships for “active” ion transport systems: II. Nonsteady-state reaction kinetic analysis of class-I mechanisms with one slow time-constant. J Membr Biol 75: 141–169.

4. Hansen U-P (1986) Reaction kinetic models of pumps, cotransporters and channels. Ion Channels and Electrogenic Pumps in Biomembranes. Abstracts of Lectures and Posters. Osaka University. pp. L13–L33.
